# Efficacy of the iron‐chelating agent, deferiprone, in patients with Parkinson's disease: A systematic review and meta‐analysis

**DOI:** 10.1111/cns.14607

**Published:** 2024-02-09

**Authors:** Ahmed Negida, Nafisa M. Hassan, Heba Aboeldahab, Youmna E. Zain, Yasmin Negida, Shirin Cadri, Nivin Cadri, Leslie J. Cloud, Matthew J. Barrett, Brian Berman

**Affiliations:** ^1^ Parkinson's and Movement Disorder Center Virginia Commonwealth University Richmond Virginia USA; ^2^ Medical Research Group of Egypt Negida Academy Arlington Massachusetts USA; ^3^ Biomedical Informatics and Medical Statistics Department, Medical Research Institute Alexandria University Alexandria Egypt; ^4^ Clinical Research Department, El‐Gomhoria General Hospital Ministry of health and population Alexandria Egypt; ^5^ Faculty of Medicine Tanta University Tanta Egypt; ^6^ Faculty of Medicine Zagazig University Zagazig Egypt; ^7^ Medical Research Group of Romania Negida Academy Arlington Massachusetts USA; ^8^ Grigore T. Popa University of Medicine and Pharmacy Iasi Romania

**Keywords:** deferiprone, iron‐chelating agent, Parkinson's disease, unified Parkinson's disease rating scale

## Abstract

**Introduction:**

Several studies have reported iron accumulation in the basal ganglia to be associated with the development of Parkinson's Disease (PD). Recently, a few trials have examined the efficacy of using the iron‐chelating agent Deferiprone (DFP) for patients with PD. We conducted this meta‐analysis to summarize and synthesize evidence from published randomized controlled trials about the efficacy of DFP for PD patients.

**Methods:**

A comprehensive literature search of four electronic databases was performed, spanning until February 2023. Relevant RCTs were selected, and their data were extracted and analyzed using the RevMan software. The primary outcome was the change in the Unified Parkinson's Disease Rating Scale (UPDRS‐III).

**Results:**

Three RCTs with 431 patients were included in this analysis. DFP did not significantly improve UPDRS‐III score compared to placebo (Standardized mean difference −0.06, 95% CI [−0.69, 0.58], low certainty evidence). However, it significantly reduced iron accumulation in the substantia nigra, putamen, and caudate as measured by T2*‐weighted MRI (with high certainty evidence).

**Conclusion:**

Current evidence does not support the use of DFP in PD patients. Future disease‐modification trials with better population selection, adjustment for concomitant medications, and long‐term follow up are recommended.

## Graphical abstract

A systematic literature review and meta‐analysis of Deferiprone compared to placebo in Parkinson's Disease patients. Current evidence showed that compared to placebo, deferiprone was not effective in improving motor functions or reducing iron deposition in brain as evident by MRI and serum ferritin.

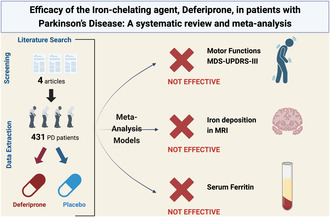

## 1 INTRODUCTION

Parkinson's disease (PD) is the second most common neurodegenerative disease worldwide. In 2020, it affected about 9.4 million of the population globally.^1^ PD is a degenerative disorder characterized by death in dopamine‐generating cells of the brain, causing motor impairment, gait disturbances, cognitive impairment, dementia, anxiety, depression, and sleep disorders. The exact pathogenesis of PD is still unknown, and till the moment, there are no disease‐modifying agents that can slow the disease progression. Historically, PD progression has been assessed by several scales such as Webster,^2^ UCLA Rating Scales,^3^ and the Unified Parkinson's Disease Rating Scale (UPDRS)^4^ which is frequently used to assess both motor and non‐motor symptom severity in PD patients.

It is known that iron homeostasis plays a crucial role in maintaining normal physiological brain functions,^5^, ^6^ where its disruption can interfere with mitochondrial functions accelerating neurodegenerative diseases.^7^ Several studies reported increased total iron in specific regions of the brain in PD patients, especially in Substantia Nigra pars compacta (SNpc), putamen, and globus pallidus.^8^ The reason for iron accumulation is not yet well understood, but several studies suggested different factors such as increased permeability of the blood–brain barrier,^9^ altered iron transport by transferrin‐TFR type 2,^10^ and mutations in genes relevant to iron transport and binding.^11^ Recent studies found that ferroptosis is involved in PD, which is an iron‐dependent cell death pathway for dopaminergic neurons.^12^

Iron deposition was also found in animal studies using MPTP(1‐methyl‐4‐phenyl‐1,2,3,6‐tetrahydropyridine), a neurotoxic animal model used to study different aspects of PD.^13^ Involvement of ferroptosis in dopaminergic cell death was confirmed in the MPTP mouse model, and its toxicity was inhibited by Ferostatin‐1(Fer‐1), a specific Ferroptosis inhibitor.^14^ Although the exact mechanism of ferroptosis in PD is not well understood, it has been hypothesized that iron‐chelating agents may slow the progress of PD both in animal models and clinical trials.^15^, ^16^, ^17^, ^18^

Deferoxamine (DFO), an iron chelator agent, was first introduced for transfusional iron overload in the early 1970s, but due to its short half‐life (20–30 min) and lack of oral activity, it initially had subpar results. To overcome these pharmacokinetics problems, Deferiprone (DFP) was introduced to clinical practice in the 1980s.^15^ DFP is a small lipophilic bidentate chelator that has good bioavailability, but the rapid biotransformation speeds up its clearance.

The effect of DFP was investigated in different neurological diseases associated with iron accumulation in the brain namely neuroferritinopathy,^19^ Friedreich Ataxia,^20^ and Pantothenate Kinase‐Associated Neurodegeneration (PKAN) disease.^21^ Results were not inclusive where DFP was beneficial in some studies,^19^, ^20^ worsened ataxia when given in high doses,^20^ and showed slight insignificant slowing of disease progression.^21^

Although DFP was initially developed to treat patients with hemoglobinopathies, it became a candidate for treating PD patients due to its ability to cross the blood–brain barrier and its neuroprotective actions through iron chelation. Its safety and efficacy in PD patients were tested in three clinical trials that were represented in four articles. The results of these clinical trials showed disagreement, where two of them (DEVOS 2014^22^ and Martin‐Bastida 2017^23^) found that DFP has a beneficial effect in treating patients with PD. On the contrary, DEVOS 2022^24^ reported that DFP has a worsening effect on PD patients. Therefore, we conducted this systematic review and meta‐analysis study to evaluate the efficacy of DFP on both motor and non‐motor symptoms in patients with PD.

## 2 METHODS

We followed the preferred reporting items of systematic reviews and meta‐analysis (PRISMA statement) guidelines when reporting this manuscript.^25^ This work was conducted in adherence to the Cochrane Handbook of Systematic Reviews of Interventions.^26^ This study was prospectively registered on PROSPERO (CRD42023396466).

### 2.1 Criteria of the considered studies

Studies satisfying the following inclusion criteria were included in the systematic review:

- Population: studies on patients with Parkinson's Disease, diagnosed according to the UK brain bank criteria or the MDS diagnostic criteria.
- Intervention: In studies where the experimental group received DFP, all doses were eligible.
- Comparator: studies where the control group received a placebo.
- Outcome: studies reporting at least the UPDRS scores and changes in MRI iron deposition in PD patients.
- Study design: studies described as randomized controlled trials where patients were assigned to the treatment groups in a random allocation method.

We excluded articles that were (1) case reports/case series, (2) thesis, (3) conference abstracts, (4) animal studies, and (5) studies on neurodegenerative diseases other than PD or neurodegeneration where the patients were not diagnosed according to the previously mentioned PD criteria.

### 2.2 Literature search and keywords

We searched PubMed, Web of Science, Cochrane, and Scopus for relevant studies in February 2023. For a sensitive search strategy, we used MESH keywords. The keywords were “Deferiprone” and “Parkinson's Disease.” The search strategy for PubMed database was: ((“Parkinson Disease”[Mesh]) AND “Deferiprone”[Mesh]) OR ((Deferiprone OR Ferriprox OR C7H9NO2) AND Parkinson's).

### 2.3 Screening and study selection process

We used Rayyan^27^ for semi‐automated screening of the literature search results. Studies were screened in two phases. The first phase was title/abstract screening for potential clinical studies. In the second phase, we retrieved the full‐text articles of the selected abstracts for further eligibility screening. Literature search and screening was done independently by two review authors (AN and HA), and any disagreements were resolved by opinion of a senior reviewer (NMH).

### 2.4 Data extraction

For all included studies, data were extracted to a uniform online data extraction sheet. Extracted data were mainly divided into four domains: (1) study characteristics, (2) characteristics of the included studies' population, (3) risk of bias domains, and (4) study outcomes.

### 2.5 Risk of Bias assessment

We assessed the risk of bias in the included studies using the Cochrane risk of bias (ROB) tool. The Cochrane ROB tool examines the potential of bias in seven study domains, including (1) random sequence generation, (2) allocation concealment, (3) blinding of the investigators and patients, (4) blinding of the outcome assessors, (5) incomplete outcome data, (6) selective outcome reporting, and (7) other sources of bias. In each domain, each study was tagged as “low risk,” “high risk,” or “unclear” after careful revision of the data presented in the published articles.

### 2.6 Publication bias

In agreement with Egger et al.,^28^ it was inapplicable to examine potential publication bias in our review via Egger's test for the funnel plot asymmetry, when the number of the included studies was less than ten studies.

### 2.7 Measures of treatment effect

Studies assessing the impact of DFP supplementation in PD patients usually provide MRI findings and laboratory values of ferritin as explanatory outcomes. The primary outcome measure for this systematic review is the Unified Parkinson's Disease Rating Scale – Part III or the old MDS‐UPDRS‐III (motor functions). Reduction in the UPDRS‐III indicates symptom improvement, while an increased score indicates worsening of the condition. Other secondary outcomes include:

1. Magnetic resonance imaging (T2*MRI) was used to quantify the change in Iron accumulation from baseline in Substantia Nigra, Caudate, and Putamen. Expressed in msec, where the increase in T2* value indicates decrease in the iron deposition in different parts of the brain.
2. Serum Ferritin indicates the change in serum iron level from baseline which reflects the ability of DFP to chelate labile iron measured in ng/mL.
3. The total MDS‐UPDRS/UPDRS score measures the progression of Parkinson's disease, with a maximum score of 199 which indicates the worst stage of PD.
4. The Mini‐Mental State Examination (MMSE) and the Montreal Cognitive Assessment (MoCA) were used to display PD patients' cognitive status. Higher scores reflect better cognition for both scales.
5. Parkinson's Disease Questionnaire (PDQ‐39) is a 39 items used to assess PD related quality of life. Its values range from 0 to 100, with higher scores indicating a worsening in the patient's life quality.
6. Adverse effects and safety outcomes are defined as the number of patients with adverse events.

### 2.8 Evidence synthesis

For studies where the UPDRS‐III/MDS‐UPDRS‐III was reported in the graph only (without numerical data), we used the graph reader web tool.^29^ In the case of absent mean and SD in the published article, we calculated them from the median and interquartile ranges according to the methods of Wan et al.^30^ To convert R*MRI to T2*, we used the following equation: R2* = 1/T2*(msˉ^1^) ×10^3^. For MRI findings, we used RevMan Online calculator^31^ to combine mean and SD of iron accumulation values in left and right of each of the following brain sites: Putamen, SN, and Caudate. Data of the MDS‐UPDRS‐III/UPDRS‐III score were pooled as the standardized mean difference (SMD) between the two study groups from baseline to endpoint with the corresponding 95% confidence intervals in a random effect model of meta‐analysis. We used the review manager RevMan^31^ version 5.3 for macOS. In the case of a delayed‐start disease‐modification trial, we restricted the data extraction and analysis to the phase where the delayed‐start group received placebo only and compared against the same treatment duration of the early start group.

### 2.9 Choice of the meta‐analysis model

We used the DerSimonian Liard method when calculating the pooled effect size for all the reported outcomes. This random model gives more weight to studies with small sample size on the expense of that with large sample size, which accommodates inconsistent effect sizes by assigning larger standard error to the pooled estimate. Therefore, these possible inconsistencies in our estimates must be taken into consideration.

### 2.10 Assessment of heterogeneity

We used Cochran Q test (chi‐square test) and Higgins and Thompson I‐squared to assess the heterogeneity among the included studies via the following equation: *I* ^2^ = ((Q‐df)/Q) ×100%. Heterogeneity was considered significant when the chi‐square test *p*‐value is less than 0.1 and the *I* ^2^ test is greater than 50%.^32^

## 3 RESULTS

### 3.1 Literature search results

**FIGURE 1:**
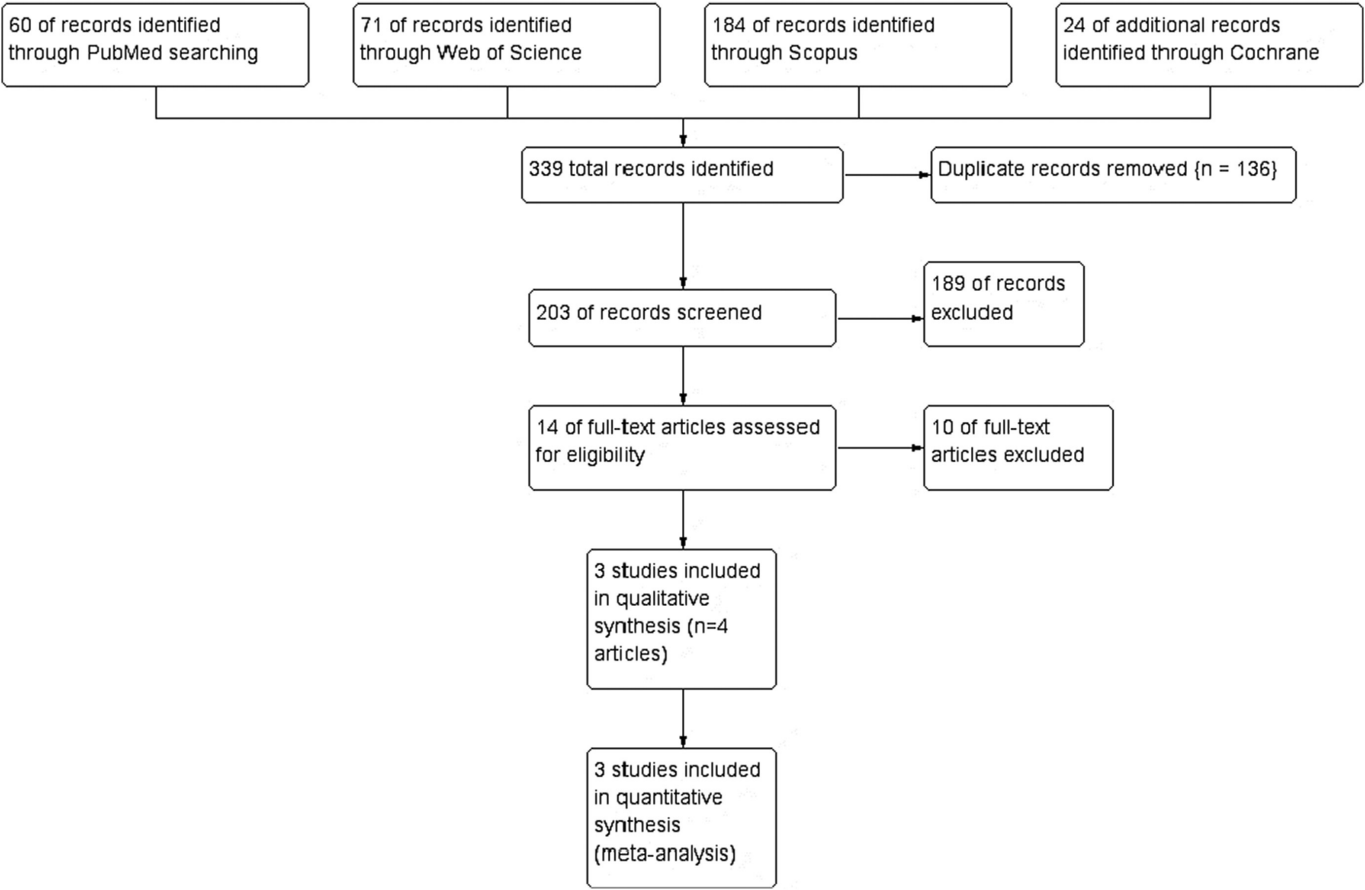
PRISMA flow diagram of the study selection process.

Three hundred thirty‐nine records were obtained from the literature search. Of them, 136 were identified by Rayyan as duplicates. After excluding irrelevant abstracts and reviews, 14 articles were eligible for full‐text screening. Of them, four articles describing three RCTs were included in this systematic review and meta‐analysis. The PRISMA flow diagram of the study selection process is shown in Figure 1.

### 3.2 Characteristics of the included studies

All studies were randomized controlled studies where PD patients were allocated to receive either the DFP or the placebo intervention. All the three studies provided DFP at a daily dose of 15 mg/kg twice daily. One study (Martin‐Bastida 2017) provided an additional dose of 10 mg/kg BID. The study of Devos (2014) started with a dose escalation period where patients received 300 mg DFP until a target dose of 30 mg/kg was achieved. The study duration ranged from 6 to 18 months. All studies recruited patients with early PD but with variable definitions of the disease stage. Devos et al. (2022) recruited patients within the first 2 years of the diagnosis without any levodopa treatment, while Martin‐Bastida et al. and Devos et al. (2014) included early PD patients who were diagnosed <5 and <3 years before enrolment in the study, respectively. Summary of the included studies and the characteristics of their populations are shown in Tables 1, and 2, respectively.

**TABLE 1:** Summary of the included studies.

Author, year (Study ID)	Location	Year	Population	Intervention	Dose	Comparator	Study duration	Outcome measures	Evaluation time points	Key findings
Devos 2014	Lille, France	2009–2011	Early‐stage stable PD <3 years from diagnosis on dopaminergic drugs (dopamine agonist and/or low doses of L‐dopa)	Oral DFP	30 mg/kg/day	Placebo	18 months	UPDRS/MRI findings	At baseline, 6, 12, and 18 months	The initial data from animal studies and pilot trial justify the need for further assessment of DFP for potential symptomatic or disease‐modification benefits.
Martin‐Bastida 2017	Lille, France	2012–2013	Early‐stage stable PD <5 years from diagnosis who were receiving PD medication regimens	Oral DFP	10 or 15 mg per kg of body weight twice daily	Placebo	6 months	UPDRS/MRI findings	At baseline, 3, and 6 months	Trend for improvement in motor‐UPDRS scores with no adverse effects on cognitive functions and mood
Devos 2022	International trial conducted at 23 sites	2016–2020	Participants with newly diagnosed Parkinson's disease who had never received levodopa	Oral DFP	15 mg per kg of body weight twice daily	Placebo	10 months	MDS‐UPDRS and its subscales/MRI findings	At baseline, and at 12, 24, 36, and 40 weeks	DFP did not improve but worsen the clinical score of early PD patients compared to placebo over a period of 36 weeks

**TABLE 2:** Summary of the characteristics of the population in the included studies.

Author, year (Study ID)	Group Name	*N*	Age (Mean, SD)	Sex (m%)	Disease duration since diagnosis	MDS‐UPDRS‐III	UPDRS‐III or MDS‐UPDRS‐III	PDQ‐39	Cognitive assessment using MoCA or MMSE	Serum Ferritin (ng/mL)
Devos 2014	Deferiprone (30 mg)	21	60.3 (9.54)	12 (57.14%)	2 (1.59) years	NA	23.9 (7)	NA	MMSE: 29 (1.6)	89 (66.22)
	Placebo	19	59.3 (8)	13 (68.42)	2 (1.6) years	NA	23 (7)	NA	MMSE: 28 (3.18)	89.66 (57.67)
Martin‐Bastida 2017	Deferiprone (20 mg)	7	68.57 (5.74)	4 (57.1%)	2.82 (1.8) years	NA	11.43 (3.47)	31.42 (27.38)	MMSE: 29.42 (0.77)	189 (62)^a^
	Deferiprone (30 mg)	7	62.85 (7.24)	5 (71.4%)	3.02 (7.11) years	NA	10.57 (3.79)	24.28 (16.64)	MMSE: 28.57 (1.61)	132 (37)^a^
	Placebo	8	64.38 (9.14)	3 (37.5)	3.54 (0.96) years	NA	11.38 (3.65)	23 (11.39)	MMSE: 29.25 (1.39)	100 (27)^a^
Devos 2022	Deferiprone	186	62 (10.1)	115 (61.8%)	109.5 (52–187) days	22 (9.3)	22.0 ± 9.3	20.5 (16)	MoCA: 27 (2.7)	NA
	Placebo	186	62.9 (9.2)	119 (63.9%)	101 (42–191) days	21.9 (9.3)	21.9 ± 9.3	20.2 (16.6)	MoCA: 27 (2.7)	NA

Abbreviations: All qualitative variables are represented as number(percentage), while quantitative variables are showed in Mean (Standard deviation).

*Note*: MDR‐UPDRS, Movement Disorder Society‐Sponsored Revision of the Unified Parkinson's Disease; MMSE, Mini‐Mental State Examination; MoCA, Montreal Cognitive Assessment; *N*, Sample size; PDQ‐39, Parkinson's Disease Questionnaire.

^a^ These values are presented as median (SEM).

### 3.3 Risk of Bias in the included studies

**FIGURE 2:**
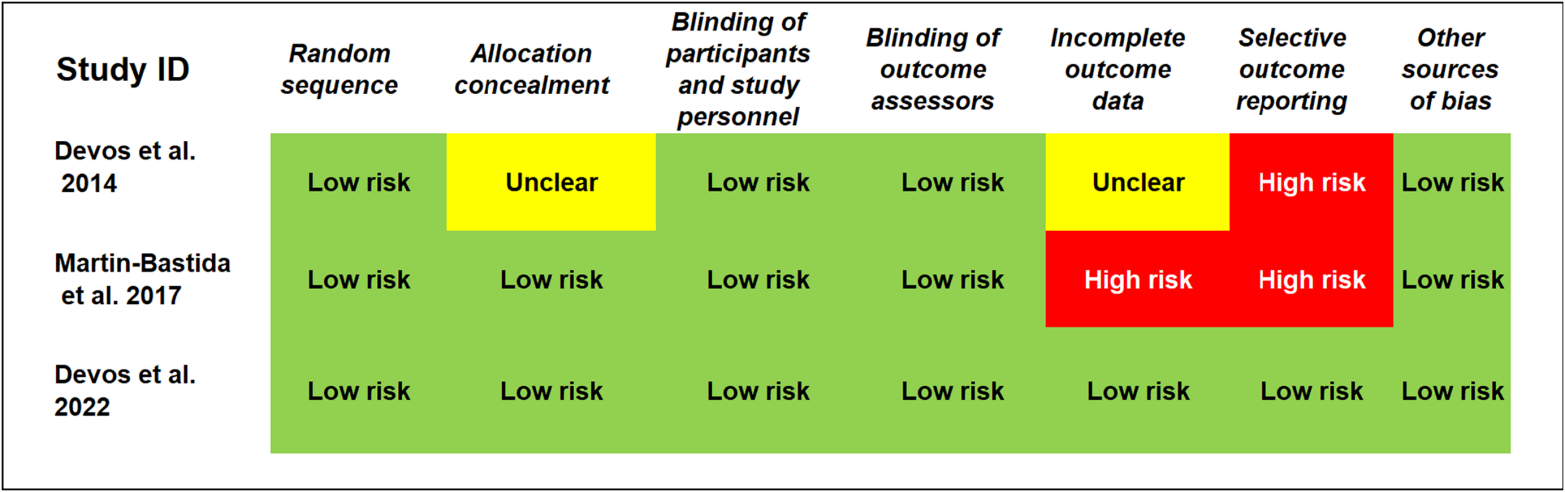
Risk of bias summary: shows review authors' judgments about each risk of bias item for each included study.

Results of the risk of bias assessment showed that the quality of included studies ranged from low to high quality. Summary of the risk of bias assessment is shown in Figure *2*. No significant risks of bias were observed in terms of the selection bias including random sequence and allocation concealment. All studies were double blinded in terms of assessment and patient treatment. Devos et al. (2014) has limited reporting of the key clinical outcomes like UPDRS total score and UPDRS‐II. Martin‐Bastida study has high risk of bias in two domains including the incomplete outcome data (the authors did not include missing patients in the analysis) and key clinical outcomes in PD patients are probably missed from reporting or were not initially measured for the study participants. Devos et al. (2022) is of high quality where all risk of bias assessment domains were of low risk.

### 3.4 Primary outcome (change in part III of the MDS‐UPDRS/UPDRS)

The overall SMD of change in the MDS/UPDRS or UPDRS‐III score did not favor either of the study groups (SMD −0.06, 95% CI [−0.69 to 0.58], *p* = 0.86, Table 3). There was significant substantial heterogeneity among the included studies (*p* = 0.04, *I* ^2^ = 70%).

**TABLE 3:** Overall SMD of change in UPDRS‐III in the included studies.

Study or subgroup	DFP			Placebo			Std. Mean Difference	
	Mean	SD	*N*	Mean	SD	*N*	Weight	IV, Random, 95% CI
Devos (2014)	−2.3	10.63	20	1	9.22	19	33.5%	−0.32 [−0.96, 0.31]
Devos (2022)	9.8	16.8	186	4	14.946	186	47.8%	0.36 [0.16, 0.57]
Martin‐Bastida (2017)	−0.115	0.31	5	0.062	0.21	8	18.7%	−0.66 [−1.81, 0.50]
Total (95% CI)			**211**			**213**	**100.00%**	**−0.06 [−0.69, 0.58]**

*Note*: Heterogeneity: Tau^2^ = 0.21; Chi^2^ = 6.66, df = 2 (*p* = 0.04); *I* ^2^ = 70%; Test for overall effect: Z = 0.18 (*p* = 0.86).

Abbreviations: CI, confidence interval; IV, inverse variance; SMD, standardized mean difference.

### 3.5 MRI changes

**FIGURE 3:**
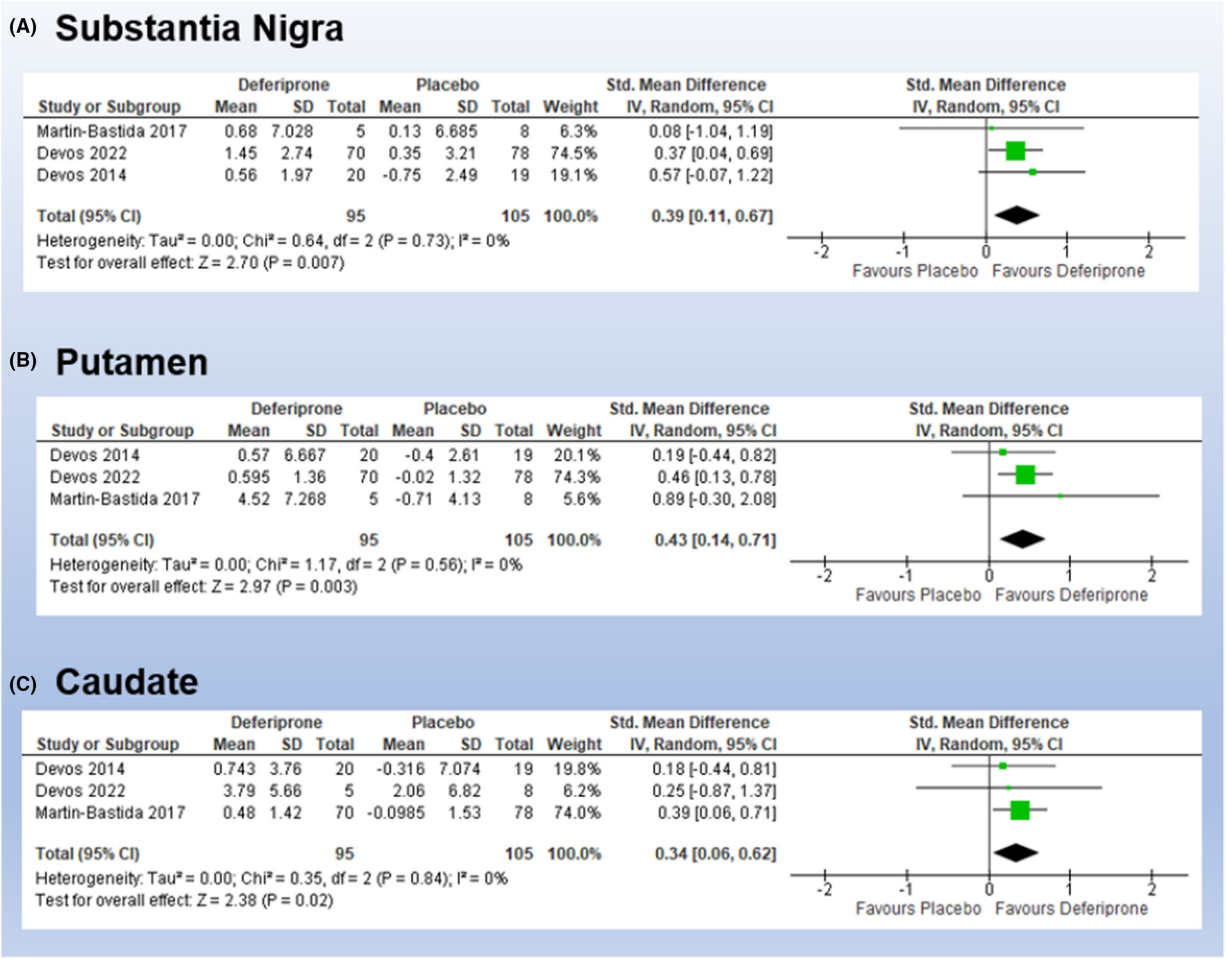
Overall SMD of MRI‐based measurements of iron load changes in (A) Substantia Nigra, (B) Putamen, and (C) Caudate. SMD, standardized mean difference.

The overall SMD of change in iron accumulation measured on T2*‐weighted MRI sequence in Substantia Nigra, Putamen, and Caudate showed a significant change in the iron load from baseline favoring the DFP group over the control, and with homogenous effect size across the pooled studies (*I* ^2^ = 0% and *p* > 0.1, for all the 3 regions) as shown in Figure 3.

### 3.6 Serum Ferritin

The pooled studies were heterogenous for the changes in serum Ferritin (*p* < 0.00001, *I* ^2^ = 96%). In the meta‐analysis, the overall SMD did not either of the two groups at (SMD −1.43, 95% CI [−3.36 to 0.50], *p* = 0.15).

### 3.7 Total MDS‐UPDRS/UPDRS scores

Devos et al. (2014) reported a reduction in the total UPDRS score within the DFP group compared to the placebo group after 6 months (difference −2.3 vs. 1) and 12 months (−1.9 vs. 1), respectively. But in the larger study Devos et al. (2022), the DFP group had more worsening of the total MDS‐UPDRS score compared to the placebo (15.6 vs. 6.3, respectively).

### 3.8 Cognitive function assessment (MoCA, MMSE)

Different scales were used to assess change in the cognitive status from baseline. This outcome was reported in only two of the included studies. Martin‐Bastida (2017) used MMSE scale, while MoCA was used by Devos (2022). Both studies reported no significant difference detected between DFP and the control group in the cognitive functions.

### 3.9 PDQ‐39

The Parkinson's disease quality of life questionnaire (PDQ‐39) results was available from two studies (Martin‐Bastida 2017, and Devos 2022). Devos (2022) reported a significant difference in the SMD of change in PDQ‐39 between the Deferiprone group versus placebo with a standardized difference in change from baseline 0.43 (95% CI, [0.18–0.68]). Although Martin observed worsening in the quality of life in the placebo and 20 mg/kg/day PD groups too, he reported a slight improvement over 6 months in the group who were administered 30 mg/kg/day without statistical significance.

### 3.10 Adverse effects and safety

Serious adverse effects under DFP were reported in the three studies. Devos (2014) stated two participants developed neutropenia at a time point of 3 and 7 months. Similarly, Martin‐Bastida (2017) reported two cases of neutropenia where a severe and rapid decline to zero cells was detected in one participant. Devos (2022) noted three participants developed Neutropenia in the DFP group versus one participant in the placebo group. Devos (2014) and (2022) reported Agranulocytosis in the DFP group where one and two participants, respectively, were detected.

Data about the adverse effect of DFP on liver functions were available from two studies Devos (2014) and Martin‐Bastida (2017). Devos et al. (2014) reported that the liver functions of all PD patients were normal at baseline and remained normal throughout the study period. But in Martin‐Bastida 2017, after 4 weeks of DFP administration, liver enzyme levels in one patient increased, then returned to normal after a 7‐day break.

In terms of safety, Devos (2014) and Martin‐Bastida (2017) stated that excluding the neutropenia incidence, DFP was well tolerated in the patients with few mild side effects. On the contrary, Devos (2022) stated that a total of 9.7% of the participants in the DFP group versus 4.8% of those in the placebo group had serious adverse effects, and a total of 87.1% of participants in the DFP group versus 80.1% of participants in the placebo group reported one or more adverse effect. In addition, Devos (2022) found general disorders and psychiatric disorders were more often reported with DFP than with the placebo, while musculoskeletal disorders were numerically fewer in the DFP group than the placebo group. Due to small sample sizes and lack of available data, statistical analysis and evidence synthesis were not possible for these outcomes.

### 3.11 GRADE assessment of the certainty in evidence

Based on GRADE assessment (Table 4), the quality of evidence on\ the changes in the MDS‐UPDRS/UPDRS‐III score (SMD −0.06) (with 95% CI from −0.69 to 0.58) was evaluated as “low.” The quality of evidence is limited by the significant heterogeneity and the imprecision resulting from the small number of included studies. In terms of the MRI‐measured reduction in iron deposition in substantia nigra, putamen, and caudate, the quality of evidence is “high.” Therefore, future studies may change our confidence in the estimate of UPDRS‐III changes but unlikely to change our confidence in the MRI‐measured iron reductions in the brain.

**TABLE 4:** GRADE assessment of the certainty in evidence in the primary outcomes.

	Risk of Bias	Inconsistencies	Indirectness	Imprecision	Publication bias	Others	Final assessment
UPDRS‐III	No	Downgrade by one level^a^	No	Downgrade by one level^b^	N/A	No	Low
MRI‐measured Iron deposition	No	No	No	No	N/A	No	High

*Note*: N/A = Not Applicable because of the small number of included studies (Egger et al.).

^a^ Owing to the significant heterogeneity in the effect estimate (*I* ^2^ = 69%).

^b^ Owing to the wide 95% CI which includes clinically important differences.

## 4 DISCUSSION

This is the first meta‐analysis to show the efficacy of DFP (an iron‐chelating agent) in PD patients. It includes 3 clinical trials (a total of 431 PD patients). Our study showed the ability of DFP to reduce iron depositions in substantia nigra, putamen, and other brain sites associated with PD progression as measured by quantitative MRI in T2*‐weighted sequences. Despite that decline in iron load, this reduction was not accompanied with any clinical improvement in motor functions of PD patients.

The disease progression was measured clinically though the total UPDRS and UPDRS‐III motor functions scores. The pooled SMD of UPDRS‐III did not favor deferiprone group; pooled studies were heterogenous in the evidence of reduction of UPDRS‐III score. This heterogeneity can be resolved by excluding Devos et al. (2022) study in the sensitivity analysis. This study has the largest sample size and highest methodological quality in the included studies. A possible explanation of heterogeneity is that the other two studies had small sample size and included PD patients while on other treatment regimens concomitant with DFP but Devos et al. (2022) patients were on DFP treatment solely and never received any dopaminergic therapy. While Devos et al. (2022) reported worsening of the total UPDRS score from baseline to week 36 in DFP as compared to placebo group, Devos et al. (2014) noticed gradual improvement in symptoms in the DFP group at 6 months in the early start group, 12 months in the delayed‐start group, 18 months, and among the last 6 months of the study to determine whether the improvement persisted throughout their study. Devos et al. (2014) interpreted the improvement as suggestive of a disease‐modifying mechanism of DFP. This highlights the need for more long‐term clinical trials that can confirm or deny their findings.

The quality of life of PD patients measured using PDQ‐39 was significantly improved in DFP group as reported in Devos (2022) which is of the highest effect of the included studies. On contrary, there is no evidence of enhancement in the cognitive functions between the deferiprone and placebo groups. Furthermore, serum ferritin didn't show significant difference between the DFP and placebo groups and showed significant heterogeneity among the included studies and this was handled through exclusion of Devos (2022) but as we said previously that this evidence is of low quality. This similar effect of DFP and placebo on serum ferritin indicates that there is no significant patients' response to iron chelation by deferiprone. The absence of benefit from DFP supplementation can be explained by the involvement of other molecular pathways in the disease. Accumulation of iron might be a factor that accelerates the progress of developing the disease but not the key trigger or regulatory step that controls the disease process.

Regarding DFP safety concerns, it must be taken into consideration that adverse effects and safety issues of the participants in the deferiprone group were detected more than those in the placebo group. Agranulocytosis is defined as an absolute neutrophil count below 500/μL, and is one of the most serious adverse effects of DFP where it occurs in 1%–2% of beta‐thalassemia patients who receive higher DFP doses (75–100 mg/kg/day).^23^ The included studies in this work collectively revealed three cases of agranulocytosis and seven cases of neutropenia (one of which reached serious zero level rapidly) where DFP was withdrawn from all cases. This necessitates the clinical monitoring of DFP administration on a weekly basis due to the high chance of side effects like agranulocytosis that may lead to discontinuation of the drug.^33^

In terms of elevated liver enzymes during DFP administration, the available data from the included studies revealed no statistical differences in liver functions at entrance versus the end of DFP treatment except in Martin‐Bastida (2017) study,^23^ which reported a 7 days discontinuation of DFP was required for liver enzymes to return to normal. According to a recent study^34^ that evaluated the long‐term safety and efficacy of DFP in the treatment of iron overload in people with Sickle cell disease and other anemias, some patients had transient increases in their ALT and AST levels. They found that four cases (3.0%) had increased ALT levels and four cases (3.0%) showed increased AST levels that could be related to DFP to the point that the treatment of two patients was interrupted due to elevated ALT levels.

Since there is no such chelator that is entirely specific for a certain metal, iron chelators would chelate other metals as well. DFP has a high affinity for zinc and could chelate clinically significant zinc pools in addition to iron. Zinc deficiency has been reported in up to 14% of patients receiving long‐term treatment with DFP suggesting the need for zinc supplementation.^35^, ^36^ On the other hand, several studies investigated serum and CSF zinc levels in PD patients unrelated to DFP administration and showed controversial findings.^37^ Other studies argued that zinc deficiency could be related to Levodopa administration in PD patients. Matsuyama et al.^38^ investigated the relationship between levodopa administration period, dosage, and zinc level in PD patients. They indicated that levodopa strongly influenced serum zinc levels which might have alleviating effects on psychiatric symptoms and recommended preventing zinc deficiency as an important step in PD treatment.

DFP showed a reduction in dopamine loss in cell lines and animal models with dopamine depletion studies. They found that it can chelate labile iron in certain brain sites lead to attenuation of the radioactive oxygen species, so it can provide protection to dopaminergic neurons and dopamine. Moreover, its ability to prevent systemic and CNS iron loss generated by other chelators by donating chelated iron to serum proteins with iron binding capacity, this increases its safety to be used clinically. This effectiveness was inspiring to researchers to try it in human.

It should be mentioned that Devos (2014) and Martin‐Bastida (2017) included PD patients with longer disease duration compared to Devos (2022). Additionally, PD patients in the first two studies were on symptomatic therapy but those in Devos (2022) were untreated. Since all patients with PD should be treated, the substantial worsening of MDS‐UPDRS scores in the two groups of Devos (2022) might be attributed to the lack of dopaminergic treatment. On the other hand, the symptomatic therapy in the studies of Devos (2014) and Martin‐Bastida (2017) may interfere with the disease‐modification effects. Although Devos (2014) was designed as a delayed‐start trial to test disease‐modification effects, we restricted the analysis to the trial duration where patients were receiving DFP or placebo but not as a delayed‐start versus early‐start since this comparison did not exist in the other two studies.

To our knowledge, this is the first comprehensive analytic approach to investigate the efficacy and safety of DFP in PD. It showed the effectiveness of DFP in declining iron deposition in the main brain areas affected in PD patients. These findings did not reveal clinical improvement in the disease progression. Our study results are limited by the small sample sizes of the included studies. In addition, they showed different points of time for assessment, unlike doses, and various degrees of the disease among the participants. This meta‐analysis shed light on the various aspects of assessing PD and raised attention to the need for more clinical trials with larger sample sizes, as well as with standardization of drug dose, patient population, and outcome assessments. In addition, it highlighted the need for more information about the safety of DFP administration especially on long‐term basis, and the necessity of clinical monitoring and proper management of the cases.

## 5 CONCLUSION

Current evidence does not support the use of DFP in PD patients. Future disease‐modification trials with better population selection, adjustment for concomitant medications, and long‐term follow up are recommended.

## CONFLICT OF INTEREST STATEMENT

The authors report no conflicts of interest in this work. This study was prospectively registered on PROSPERO (CRD42023396466).

## Data Availability

Data used in this study are available upon a request from the corresponding author.

## References

[1] Maserejian N , Vinikoor‐Imler L , Dilley A . Estimation of the 2020 Global Population of Parkinson's Disease (PD) [Abstract]. MDS Abstracts. Accessed February 3, 2023. https://www.mdsabstracts.org/abstract/estimation‐of‐the‐2020‐global‐population‐of‐parkinsons‐disease‐pd/

[2] Webster DD . Critical analysis of the disability in Parkinson's disease. Mod Treat. 1968;5(2):257‐282. 5655944

[3] Diamond SG , Markham CH , Treciokas LJ . A double‐blind comparison of levodopa, madopa, and sinemet in Parkinson disease. Ann Neurol. 1978;3(3):267‐272. doi:10.1002/ana.410030314 352236

[4] Goetz CG , Tilley BC , Shaftman SR , et al. Movement Disorder Society‐sponsored revision of the unified Parkinson's disease rating scale (MDS‐UPDRS): scale presentation and clinimetric testing results. Mov Disord off J Mov Disord Soc. 2008;23(15):2129‐2170. doi:10.1002/mds.22340 19025984

[5] Dusek P , Hofer T , Alexander J , Roos PM , Aaseth JO . Cerebral iron deposition in neurodegeneration. Biomolecules. 2022;12(5):714. doi:10.3390/biom12050714 35625641 PMC9138489

[6] Ward RJ , Zucca FA , Duyn JH , Crichton RR , Zecca L . The role of iron in brain ageing and neurodegenerative disorders. Lancet Neurol. 2014;13(10):1045‐1060. doi:10.1016/S1474-4422(14)70117-6 25231526 PMC5672917

[7] Horowitz MP , Greenamyre JT . Mitochondrial iron metabolism and its role in neurodegeneration. J Alzheimers Dis. 2010;20(s2):S551‐S568. doi:10.3233/JAD-2010-100354 20463401 PMC3085540

[8] Jellinger K , Paulus W , Grundke‐Iqbal I , Riederer P , Youdim MBH . Brain iron and ferritin in Parkinson's and Alzheimer's diseases. J Neural Transm Park Dis Dement Sect. 1990;2(4):327‐340. doi:10.1007/BF02252926 2078310

[9] Kortekaas R , Leenders KL , van Oostrom JCH , et al. Blood–brain barrier dysfunction in parkinsonian midbrain in vivo. Ann Neurol. 2005;57(2):176‐179. doi:10.1002/ana.20369 15668963

[10] Mastroberardino PG , Hoffman EK , Horowitz MP , et al. A novel transferrin/TfR2‐mediated mitochondrial iron transport system is disrupted in Parkinson's disease. Neurobiol Dis. 2009;34(3):417‐431. doi:10.1016/j.nbd.2009.02.009 19250966 PMC2784936

[11] Guerreiro RJ , Bras JM , Santana I , et al. Association of HFE common mutations with Parkinson's disease, Alzheimer's disease and mild cognitive impairment in a Portuguese cohort. BMC Neurol. 2006;6(1):24. doi:10.1186/1471-2377-6-24 16824219 PMC1534050

[12] Stockwell BR , Friedmann Angeli JP , Bayir H , et al. Ferroptosis: a regulated cell death nexus linking metabolism, redox biology, and disease. Cell. 2017;171(2):273‐285. doi:10.1016/j.cell.2017.09.021 28985560 PMC5685180

[13] Jackson‐Lewis V , Blesa J , Przedborski S . Animal models of Parkinson's disease. Parkinsonism Relat Disord. 2012;18:S183‐S185. doi:10.1016/S1353-8020(11)70057-8 22166429

[14] Do Van B , Gouel F , Jonneaux A , et al. Ferroptosis, a newly characterized form of cell death in Parkinson's disease that is regulated by PKC. Neurobiol Dis. 2016;94:169‐178. doi:10.1016/j.nbd.2016.05.011 27189756

[15] Ward RJ , Dexter DT , Martin‐Bastida A , Crichton RR . Is chelation therapy a potential treatment for Parkinson's disease? Int J Mol Sci. 2021;22(7):3338. doi:10.3390/ijms22073338 33805195 PMC8036775

[16] Kaur D , Yantiri F , Rajagopalan S , et al. Genetic or pharmacological iron chelation prevents MPTP‐induced neurotoxicity in vivo: a novel therapy for Parkinson's disease. Neuron. 2003;37(6):899‐909. doi:10.1016/S0896-6273(03)00126-0 12670420

[17] AAlikhani M , Khalili M , Jahanshahi M . The natural iron chelators' ferulic acid and caffeic acid rescue mice's brains from side effects of iron overload. Front Neurol. 2022;13:951725. doi:10.3389/fneur.2022.951725 36313492 PMC9614107

[18] Perez CA , Tong Y , Guo M . Iron chelators as potential therapeutic agents for Parkinson's disease. Curr Bioact Compd. 2008;4(3):150‐158. doi:10.2174/157340708786305952 19809592 PMC2756717

[19] Marchand F , Moreau C , Kuchcinski G , Huin V , Defebvre L , Devos D . Conservative iron chelation for neuroferritinopathy. Mov Disord. 2022;37(9):1948‐1952. doi:10.1002/mds.29145 35996824 PMC10360136

[20] Elincx‐Benizri S , Glik A , Merkel D , et al. Clinical experience with deferiprone treatment for Friedreich ataxia. J Child Neurol. 2016;31(8):1036‐1040. doi:10.1177/0883073816636087 27029487

[21] Klopstock T , Tricta F , Neumayr L , et al. Safety and efficacy of deferiprone for pantothenate kinase‐associated neurodegeneration: a randomised, double‐blind, controlled trial and an open‐label extension study. Lancet Neurol. 2019;18(7):631‐642. doi:10.1016/S1474-4422(19)30142-5 31202468

[22] Devos D , Moreau C , Devedjian JC , et al. Targeting chelatable iron as a therapeutic modality in Parkinson's disease. Antioxid Redox Signal. 2014;21(2):195‐210. doi:10.1089/ars.2013.5593 24251381 PMC4060813

[23] Martin‐Bastida A , Ward RJ , Newbould R , et al. Brain iron chelation by deferiprone in a phase 2 randomised double‐blinded placebo controlled clinical trial in Parkinson's disease. Sci Rep. 2017;7(1):1398. doi:10.1038/s41598-017-01402-2 28469157 PMC5431100

[24] Devos D , Labreuche J , Rascol O , et al. Trial of deferiprone in Parkinson's disease. N Engl J Med. 2022;387(22):2045‐2055. doi:10.1056/NEJMoa2209254 36449420

[25] Page MJ , McKenzie JE , Bossuyt PM , et al. The PRISMA 2020 statement: an updated guideline for reporting systematic reviews. Int J Surg. 2021;88:105906. doi:10.1016/j.ijsu.2021.105906 33789826

[26] Higgins JPT , Thomas J , Chandler J , et al. Cochrane Handbook for Systematic Reviews of Interventions. John Wiley & Sons; 2019.

[27] Rayyan—a web and mobile app for systematic reviews | SpringerLink. Accessed March 10, 2023. doi:10.1186/s13643-016-0384-4

[28] Egger M , Smith GD , Schneider M , Minder C . Bias in meta‐analysis detected by a simple, graphical test. BMJ. 1997;315(7109):629‐634. doi:10.1136/bmj.315.7109.629 9310563 PMC2127453

[29] Larsen K . Graphreader. http://www.graphreader.com

[30] Estimating the sample mean and standard deviation from the sample size, median, range and/or interquartile range | SpringerLink. Accessed March 10, 2023. doi:10.1186/1471-2288-14-135 PMC4383202 25524443

[31] Review Manager (RevMan) [Computer program]. Version 5.4. The Cochrane Collaboration. 2020.

[32] Higgins JPT , Thompson SG , Deeks JJ , Altman DG . Measuring inconsistency in meta‐analyses. BMJ. 2003;327(7414):557‐560. doi:10.1136/bmj.327.7414.557 12958120 PMC192859

[33] Kontoghiorghes GJ , Kontoghiorghe CN . Efficacy and safety of iron‐chelation therapy with deferoxamine, deferiprone, and deferasirox for the treatment of iron‐loaded patients with non‐transfusion‐dependent thalassemia syndromes. Drug Des Devel Ther. 2016;10:465‐481. doi:10.2147/DDDT.S79458 PMC4745840 26893541

[34] Elalfy MS , Hamdy M , El‐Beshlawy A , et al. Deferiprone for transfusional iron overload in sickle cell disease and other anemias: open‐label study of up to 3 years. Blood Adv. 2023;7(4):611‐619. doi:10.1182/bloodadvances.2021006778 36018224 PMC9979751

[35] al‐Refaie FN , Wonke B , Wickens DG , Aydinok Y , Fielding A , Hoffbrand AV . Zinc concentration in patients with iron overload receiving oral iron chelator 1,2‐dimethyl‐3‐hydroxypyrid‐4‐one or desferrioxamine. J Clin Pathol. 1994;47(7):657‐660. doi:10.1136/jcp.47.7.657 8089225 PMC502110

[36] Maclean KH , Cleveland JL , Porter JB . Cellular zinc content is a major determinant of iron chelator–induced apoptosis of thymocytes. Blood. 2001;98(13):3831‐3839. doi:10.1182/blood.V98.13.3831 11739193

[37] Sikora J , Ouagazzal AM . Synaptic zinc: an emerging player in Parkinson's disease. Int J Mol Sci. 2021;22(9):4724. doi:10.3390/ijms22094724 33946908 PMC8125092

[38] Matsuyama H , Matsuura K , Ishikawa H , et al. Correlation between serum zinc levels and levodopa in Parkinson's disease. Nutrients. 2021;13(11):4114. doi:10.3390/nu13114114 34836369 PMC8621473

